# Changes Associated with the Peri-Ovulatory Period, Age and Pregnancy in ACTH, Cortisol, Glucose and Insulin Concentrations in Mares

**DOI:** 10.3390/ani11030891

**Published:** 2021-03-20

**Authors:** Gemma R. Hicks, Natalie S. Fraser, François-René Bertin

**Affiliations:** 1 School of Veterinary Science, The University of Queensland, Gatton, QLD 4343, Australia; g.hicks@uq.edu.au (G.R.H.); matickan@vt.edu (N.S.F.); 2 Samford Valley Veterinary Hospital, Samford, QLD 4520, Australia

**Keywords:** early pregnancy, endocrinology, equine, hypothalamic–pituitary–adrenal axis, insulin dysregulation, metabolism, oestrous cycle, pituitary pars intermedia dysfunction

## Abstract

**Simple Summary:**

The reproductive cycle of mares is associated with many hormonal changes, but the effects of this cycle and pregnancy on adrenocorticotropic hormone and insulin concentrations are poorly described, which could limit our ability to diagnose pituitary pars intermedia dysfunction and insulin dysregulation. In this study, healthy mares are followed through their reproductive cycle during the physiologic breeding season, and adrenocorticotropic hormone, cortisol, glucose and insulin concentrations are measured to determine the effects of age, pregnancy and the reproductive cycle on analyte concentrations. No significant effect of age, the reproductive cycle or pregnancy is detected on the cortisol, glucose or insulin concentrations; however, adrenocorticotropic hormone concentrations are significantly increased throughout the peri-ovulatory period and during pregnancy. Therefore, knowledge of a mare’s reproductive cycle might be beneficial when interpreting adrenocorticotropic hormone concentrations.

**Abstract:**

Although there are many hormonal changes associated with reproduction, the effects of ovulation and early pregnancy on adrenocorticotropic hormone (ACTH) and insulin concentrations are poorly described. We hypothesise that both ovulation and early pregnancy will alter ACTH and insulin concentrations in healthy mares. Eighteen mares showing no clinical signs suggestive of, or laboratory findings consistent with, pituitary pars intermedia dysfunction PPID and insulin dysregulation (ID) are enrolled. ACTH, cortisol, insulin and glucose concentrations are measured over their peri-ovulatory period, as determined via ultrasounds and progesterone concentrations. The mares are grouped by age and gestation status, and a two-way repeated-measures ANOVA is used to determine the effects of age and early pregnancy, along with the peri-ovulatory period, on analyte concentrations. No significant effect of age, ovulation or early pregnancy is detected on the mares’ cortisol, insulin or glucose concentrations; however, there is a significant effect of early pregnancy and ovulation on ACTH concentrations (*p* = 0.04 and *p* = 0.04 respectively). ACTH concentrations change around ovulation and with early pregnancy. Therefore, knowledge of a mare’s reproductive status might be beneficial when interpreting ACTH concentrations.

## 1. Introduction

The hypothalamo–pituitary–adrenocortical (HPA) axis is the main control of the neurological and endocrine mechanisms, as it integrates external and internal stimuli. Several studies have identified the role of external environmental factors, such as season or diet, on adrenocorticotropic hormone (ACTH) secretion, as well as the role of critical illness on the HPA axis’ function [1,2,3]. There are, however, limited data available regarding the role of physiological internal factors. Mares are seasonally polyoestrous breeders and undergo neuroendocrine changes throughout their peri-ovulatory period [4]. The mare’s cycle is a reflection of changes to multiple external and internal factors, such as days’ length, age, ovarian capacity and disease status [4]. The investigation of endocrinopathies in mares showing signs of reproductive failure—such as infertility, irregular peri-ovulatory periods or chronic uterine infections—is recommended; however, the effect of ovulation on this commonly measured hormone is poorly described.

Pituitary pars intermedia dysfunction (PPID) is the most common endocrinopathy of older horses, primarily affecting the HPA axis [5,6]. The pathophysiology of the disease is not completely understood; however, PPID is commonly observed in horses older than 18–20 years of age [5]. One of the most debilitating consequences of PPID is laminitis. Although the link between laminitis and PPID is not clear, between 30 and 70% of horses with PPID are also diagnosed with insulin dysregulation (ID) [7,8]. These two conditions are different, but they are connected, and the presence of ID in horses with PPID is associated with laminitis [9]. Equine ID is described as a complex metabolic disorder, consisting of both peripheral tissue insulin resistance and hyperinsulinemia (basal or post-prandial), with hyperinsulinemia being associated with laminitis development [10]. Similar to that which has been described for PPID, previous studies have demonstrated how insulin sensitivity varies with environmental and pathological factors, such as seasonal changes, stress, age, genetics, diet, obesity and systemic disease, but limited data are available regarding the physiological role of internal factors [11,12,13,14,15].

Therefore, the aims of the study are to determine the physiological changes in ACTH and insulin concentrations in mares going through ovulation and to investigate the effects of early pregnancy in order to understand if these changes could have an impact on a diagnosis of PPID or ID.

## 2. Materials and Methods

Eighteen mares with a median age of 10 years old (ranging from 3 to 18 years old)—consisting of a combination of Stockhorse (n = 8), Standardbred (n = 5) and Thoroughbred (n = 5) horses—were followed daily and multiple times a day when close to ovulation during the physiologic breeding season, and they were enrolled in the study. A physical examination, including body condition score (BCS) and cresty neck score (CNS), was performed on each mare before enrolment, and all were considered healthy [16,17]. None of the mares exhibited clinical signs suggestive of PPID or ID (e.g., hypertrichosis or delayed shedding, epaxial muscle wastage, abnormal sweating, abnormal fat distribution or laminitis) nor laboratory findings consistent with those diseases (ACTH within seasonally adjusted diagnostic cut-off values [3] and baseline immunoreactive serum insulin concentration <20 µIU/mL [18]). The housing conditions and diets were the same for all mares, which were kept together in large, rotational paddocks with an unlimited supply of fresh water and lucerne hay provided by the same people. The mares were part of a reproduction teaching herd and thus were used to being manipulated without sedation. The use of animals and the experimental design of this study were approved by the Institutional Animal Ethics Committee (approval number: SVS/332/19).

Routine ultrasound examinations were performed daily between September and November (southern hemisphere) by a board-certified theriogenologist (NSF) to identify the stage of the mare’s peri-ovulatory period. Progesterone concentrations were also measured to confirm the peri-ovulatory period status. The mares were inseminated with fresh semen within 24 h of ovulation from stallions of known fertility, and ongoing ultrasound examinations were performed to identify pregnancy or no pregnancy. Blood samples were obtained less than 8 h before ovulation (pre-ovulatory period), at insemination (postovulatory period) and at the 14-day early pregnancy diagnosis (EPD) by means of jugular venepuncture. The samples were placed into 10 mL ethylenediaminetetraacetic (EDTA) and a 10 mL serum separator tube (SST) (Vacutainer Tubes, BD, Belliver Industrial Estate, Plymouth, PL6 7BP, UK). The EDTA and SST tubes were centrifuged at 4 °C for 10 min at 1574 g to separate the plasma and serum.

After centrifugation, the plasma and serum samples were transferred via pipette to 1.5 mL polypropylene tubes and stored in a −80 °C freezer until analysis. Chemiluminescent assays were used to measure the immunoreactive plasma ACTH, immunoreactive serum cortisol, immunoreactive serum progesterone and immunoreactive serum insulin concentrations, as previously described (Immulite 1000 Chemiluminescent Assay, Siemens, Bayswater, VIC 3153, Australia) [19,20]. The ACTH assay used to measure immunoreactive plasma ACTH concentration has been validated for use in horses, has a range of detection of 10–1250 pg/mL and has intra-assay and inter-assay variabilities of 5.4 and 4.8%, respectively [19]. Whole blood glucose concentrations were analysed immediately after blood collection using a hand-held glucometer (Alpha TRAX 2) (Alpha TRAK^®^ 2 Veterinary Blood Glucose Monitoring System, Zoetis Inc., Kalamazoo MI 49007, USA), as previously described [21].

The mares were grouped by pregnancy status (pregnant vs. non-pregnant) and age (younger or older than 12 years of age) against their peri-ovulatory period (pre-ovulatory, post-ovulatory and 14-day EPD). The data were analysed for a normal distribution using a Shapiro–Wilk test; normally distributed data are presented as mean ± standard deviation, while non-normally distributed data are presented as the median and range. The effects of the peri-ovulatory period and early pregnancy or peri-ovulatory period and age upon ACTH, cortisol, insulin and glucose concentrations were assessed using a two-way repeated-measures analysis of variance (2-way RM-ANOVA). Statistical analysis was conducted using commercially available software (Graph Pad Prism, Prism, Graph Pad Software, Inc. La Jolla, CA 92037, USA), and *p* < 0.05 was considered statistically significant.

## 3. Results

Of the 18 mares, 11 became pregnant, including 9 mares younger than 12 years of age and 2 mares older than 12 years of age. Six of the 18 mares were older than 12 years of age, including 2 pregnant mares and 4 non-pregnant mares. The median BCS was 5/9 (ranging from 4/9 to 6/9). Considering the narrow range in BCS, no further analysis was performed on this variable.

### 3.1. Effect of Early Pregnancy and Peri-Ovulatory Period

**Figure 1:**
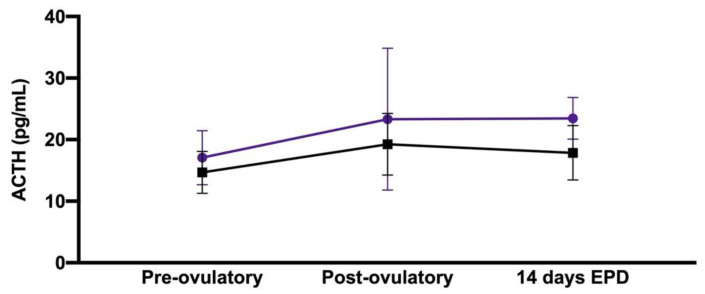
Mean ± standard deviation for immunoreactive plasma ACTH concentrations associated with early pregnancy and peri-ovulatory period, showing a significant effect of early pregnancy and time in the peri-ovulatory period upon the immunoreactive plasma ACTH concentration (*p* = 0.04 and *p* = 0.04, respectively). Purple discs indicate pregnant mares (n = 11), and black squares indicate non-pregnant mares (n = 7). The *x*-axis does not reflect the time scale.

There were significant effects of early pregnancy and time in the peri-ovulatory period upon the immunoreactive plasma ACTH concentration (*p* = 0.04 and *p* = 0.04, respectively), with higher concentrations in pregnant mares and at 14 days after ovulation; however, no post hoc comparison reached statistical significance (Table 1 and Figure 1). Meanwhile, there was no significant effect of early pregnancy and time in the peri-ovulatory period upon immunoreactive serum cortisol, immunoreactive serum insulin or blood glucose concentrations (data not shown).

### 3.2. Effect of Age and Peri-Ovulatory Period

**Figure 2:**
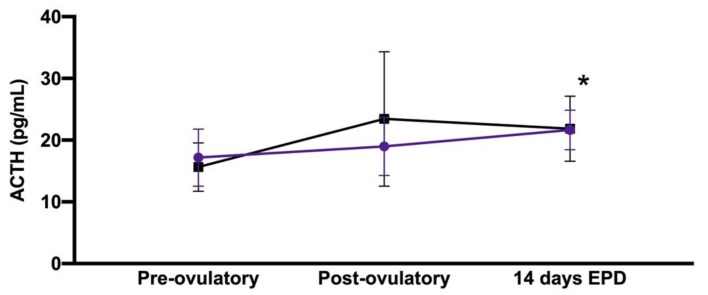
Mean ± standard deviation for immunoreactive plasma ACTH concentrations associated with age and peri-ovulatory period, showing a significant effect of time in the peri-ovulatory period (*p* = 0.04). Purple discs indicate mares ≥ 12 years of age (n = 6), and black squares indicate mares <12 years of age (n = 12). * *p* < 0.05 14-day EPD vs. pre-ovulatory, regardless of the age of the mare. The *x*-axis does not reflect the time scale.

No significant effect of age upon immunoreactive plasma ACTH concentration (*p* = 0.55) was detected, but there was a significant effect of time in the peri-ovulatory period (*p* = 0.04), with significantly higher concentrations at 14 days after ovulation compared to in the pre-ovulatory period (*p* = 0.04, Table 2 and Figure 2). Meanwhile, there was no significant effect of age and time in the peri-ovulatory period upon immunoreactive serum cortisol, immunoreactive serum insulin or blood glucose concentrations (data not shown).

## 4. Discussion

The main results of this study are that (1) immunoreactive plasma ACTH concentration is altered throughout the duration of a mare’s peri-ovulatory period and that (2) early pregnancy results in a higher immunoreactive plasma ACTH concentration.

The changes in ACTH concentration during the peri-ovulatory period are mild and unlikely to change a diagnosis of PPID; therefore, differences in immunoreactive plasma ACTH concentrations must be interpreted with caution. These changes in ACTH concentration are consistent with a recent study that investigated mares’ peri-ovulatory period with frequent sampling, showing that ACTH increased at the time of ovulation [22]. In that study, increases in ACTH were associated with increases in both cortisol and aldosterone. The measure of aldosterone was beyond the scope of our study, and the study failed to detect significant changes in cortisol concentrations. An absence of correlation between ACTH and cortisol has previously been described in different conditions beyond pregnancy or the diagnosis of PPID, with factors such as hypoglycaemia, stress, exercise and disease more likely to result in changes in cortisol secretion [2,23,24]. Although the mares included in our study did not bear any evidence of disease or stress, it is possible that the variation in cortisol concentrations associated with our low sample size prevented us from observing a significant difference. In rats, gonadal–HPA axis interactions have been described with the modulation of different levels of the HPA axis, with ovarian hormones’ oestradiol administration increasing both ACTH and cortisol concentrations [25]. In mares, however, no significant alteration of the HPA axis has been observed after ovariectomy, and oestradiol would not have had any stimulatory role on the adrenal gland [26,27]. Taken together, these data suggest that, unlike other species, for horses, the reproductive system is not as relevant in the control of the HPA axis.

This difference could be due to the proportionally larger amount of oestrogens secreted by the equine adrenal gland, as horses are the only domestic animal that can achieve oestrus after ovariectomy [28]. On the other hand, a direct effect of the equine HPA axis on the reproductive system is also possible, as ACTH administration to healthy mares resulted in increased ovarian hormonal secretion [27,29]. Unfortunately, oestradiol and luteinising hormones were not measured in this study, which would have allowed a better assessment of the possible cross-talk between the HPA axis and the reproductive system. As such, further research is warranted to better characterise equine gonadal–HPA axis interactions.

Additional possible causes that may explain some of the observed variation could be the physiological circannual and circadian changes. The circannual rhythm affects ACTH concentrations, as the activity of the HPA axis increases in late summer and autumn, leading to higher ACTH concentrations [3]. These changes demonstrate the importance of implementing seasonally adjusted diagnostic cut-off values to ensure ACTH concentrations are interpreted appropriately in horses suspected of PPID [3]. The impact of the seasons in this study can, however, be considered to be limited since the samples were collected in early spring, a time considered to be a quiescent phase with limited variations in ACTH concentrations. The circadian rhythm could be more relevant in this study, as the mares did not all ovulate at the same time. Although these changes are also unlikely to lead to misdiagnoses of PPID, they could explain some of the changes observed in the current study, with higher immunoreactive plasma ACTH concentrations in the morning [30].

Pregnancy has been shown to increase the HPA axis’ activity. For example, referring to previous research, in pregnant Spanish Purebred and Standardbred mares, dopamine, ACTH, testosterone and cortisol increased with pregnancy [31,32,33]. This is partially consistent with our findings, as only ACTH, and not cortisol, increased during the study. Interestingly, the increase in ACTH was only observed in the early stages of pregnancy, until the third month, while the increase in cortisol was only observed after 5 months of pregnancy [32]. The discrepancy observed could, therefore, be due to the different timings considered in the two studies. As mentioned above, an absence of correlation between ACTH and cortisol is frequently reported in horses, and cortisol is less frequently measured in clinical practice given its larger variability. Some authors have suggested that a possible explanation for the increase in ACTH concentration in early pregnancy could be the secretion of corticotropin-releasing hormone (CRH), triggered when the developing placenta stimulates maternal ACTH secretion [32,34]. Further studies have, however, failed to confirm this hypothesis as no increase in CRH has been detected in pregnant mares, possibly due to differences in placentation [35]. Although the changes in immunoreactive plasma ACTH concentrations observed in this study are above the intra- and inter-assay coefficient of variation for the ACTH assay, they are mild and unlikely to be clinically relevant for detecting early PPID. That being said, those changes might confound diagnostic testing interpretation when following-up a PPID mare during her pregnancy, or might suggest that an increase in pergolide treatment could be warranted to limit pregnancy-induced HPA axis stimulation.

Interestingly, in this study, no effect of the time in the peri-ovulatory period, early pregnancy or age was detected upon immunoreactive serum insulin or blood glucose concentration. This contradicts other studies in which age, pregnancy and season are common factors shown to have an effect on insulin secretion [14,36,37]. Several studies have identified an increase in insulin concentration in aged mares when compared to younger mares, and, although no clear mechanism has been demonstrated, this effect could be attributed to being the compensation for a decrease in peripheral tissue insulin sensitivity [36]. Both insulin secretion and sensitivity have been shown to be altered during pregnancy, with decreased peripheral tissue glucose uptake, possibly to ensure an adequate supply of nutrients is delivered to the foetus [38]. Insulin concentrations can also be affected by seasonal changes in healthy horses, with increased insulin concentrations noted during spring when compared to winter [39].

Beyond the small sample size, which has limited our ability to detect the effects demonstrated in other studies, the main limitations of the study were the lack of dynamic testing—required to better explore the HPA axis and the insulin–glucose dynamics—and the lack of frequent sampling. It has been shown that dynamic testing is more sensitive to detect small changes in pituitary activity or insulin secretion. Performing a thyrotropin-releasing hormone stimulation test, an oral glucose test or a two-step insulin sensitivity test could have improved our assessment of the endocrine status of the mares [40,41,42,43]. In addition, including mares with PPID, ID or both disorders would have allowed us to identify and validate any significant changes in hormone concentrations among healthy and metabolic mares. In the future, healthy mares could be used as a control to compare the differences in ACTH and insulin concentrations among healthy and metabolic mares in order to highlight more unique and significant changes in hormone concentrations. Finally, it must be noted that our study timeframe was short; carrying out sample collection and data analysis over a longer period could have potentially shown significant differences in hormone concentrations throughout multiple peri-ovulatory periods and complete pregnancies.

## 5. Conclusions

ACTH concentrations change during a mare’s peri-ovulatory period and early pregnancy. Therefore, in addition to the seasonal diagnostic cut-off values, the interpretation of an immunoreactive plasma ACTH concentration should take into account the clinical context of a horse, including its reproductive status.

## Figures and Tables

**Table 1:** Mean ± standard deviation for immunoreactive plasma adrenocorticotropic hormone (ACTH) concentrations associated with early pregnancy and peri-ovulatory period. (EPD: early pregnancy diagnosis).

Peri-Ovulatory Period	Pregnant Mares (n = 11)	Non-Pregnant Mares (n = 7)
Pre-ovulatory	17.1 + 4.4 pg/mL	14.7 + 3.4 pg/mL
Post-ovulatory	23.3 + 11.5 pg/mL	19.2 + 5.0 pg/mL
14-day EPD	23.5 + 3.4 pg/mL	17.9 + 4.4 pg/mL

**Table 2:** Mean ± standard deviation in immunoreactive plasma adrenocorticotropic hormone (ACTH) concentrations associated with age and peri-ovulatory period. (EPD: early pregnancy diagnosis).

Peri-Ovulatory Period	Older Mares (n = 6)	Younger Mares (n = 12)
Pre-ovulatory ^a^	17.2 + 4.6 pg/mL	15.6 + 3.9 pg/mL
Post-ovulatory	19.0 + 4.7 pg/mL	23.4 + 10.9 pg/mL
14-day EPD ^a^	21.7 + 3.2 pg/mL	21.9 + 5.2 pg/mL

^a^*p* < 0.05 14 days EPD vs. pre-ovulatory, regardless of the age of the mare.

## Data Availability

The data presented in this study are available on request to the corresponding author. The data are not publicly available due to institution intellectual property management protocol.
